# Identification of hub genes and construction of transcriptional regulatory network for the progression of colon adenocarcinoma hub genes and TF regulatory network of colon adenocarcinoma

**DOI:** 10.1002/jcp.29067

**Published:** 2019-10-14

**Authors:** Shuxun Wei, Jinshui Chen, Yu Huang, Qiang Sun, Haolu Wang, Xiaowen Liang, Zhiqian Hu, Xinxing Li

**Affiliations:** ^1^ Department of General Surgery, Changzheng Hospital The Second Military Medical University Shanghai China; ^2^ The University of Queensland Diamantina Institute The University of Queensland Woolloongabba Queensland Australia

**Keywords:** colon adenocarcinoma, hub genes, Gene Ontology and Kyoto Encyclopedia of Genes and Genomes, transcription factors regulatory network, weighted gene co‐expression network

## Abstract

The aim of this study was to identify key genes related to the progression of colon adenocarcinoma (COAD), and to investigate the regulatory network of hub genes and transcription factors (TFs). Dataset GSE20916 including 44 normal colon, 55 adenoma, and 36 adenocarcinoma tissue samples was used to construct co‐expression networks via weighted gene co‐expression network. Gene Ontology annotation and the Kyoto Encyclopedia of Genes and Genomes pathway enrichment analysis for the objective module were performed using the online Database for Annotation, Visualization and Integrated Discovery. Hub genes were identified by taking the intersection of differentially expressed genes between dataset GSE20916 and GSE39582 and validated using The Cancer Genome Atlas (TCGA) database. The correlations between microRNA (miRNA) and hub genes were analyzed using the online website StarBase. Cytoscape was used to establish a regulatory network of TF‐miRNA‐target gene. We found that the orange module was a key module related to the tumor progression in COAD. In datasets GSE20916 and GSE39582, a total of eight genes (*BGN, SULF1, COL1A1, FAP, THBS2, CTHRC1, COL5A2*, and *COL1A2*) were selected, which were closely related with patients’ survivals in TCGA database and dataset GSE20916. COAD patients with higher expressions of each hub gene had a worse prognosis than those with lower expressions. A regulatory network of TF‐miRNA‐target gene with 144 TFs, 26 miRNAs, and 7 hub genes was established, including model KLF11‐miR149‐*BGN*, TCEAL6‐miR29B2‐*COL1A1*, and TCEAL6‐miR29B2‐*COL1A2*. In conclusion, during the progression of COAD, eight core genes (*BGN, SULF1, COL1A1, FAP, THBS2, CTHRC1, COL5A2*, and *COL1A2*) play vital roles. Regulatory networks of TF‐miRNA‐target gene can help to understand the disease progression and optimize treatment strategy.

## Graphical abstract

Hub genes and transcription factor regulatory network of colon adenocarcinoma

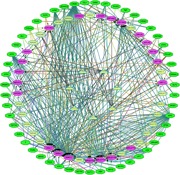

**Abbreviations**
DEGs: the differentially expressed genes
GO: Gene Ontology
KEGG: Kyoto Encyclopedia of Genes and Genomes
TCGA: The Cancer Genome Atlas
WGCNA: weighted gene co‐expression network analysis

## 1 INTRODUCTION

Colon cancer is one of the most common gastrointestinal malignancies worldwide (Arnold et al., 2017). Every year, the incidence of colorectal cancer increases by 4.2% globally (Chen et al., 2016; Maley et al., 2017). Currently, most patients with colorectal cancer cannot be diagnosed at early stages. Thus, early diagnosis and intervention are important to improve the prognosis of colorectal cancer. Colon adenocarcinoma (COAD) is the most common type of colorectal cancer (Mutch, 2007). The progression of COAD is through a multi‐stage process, from normal mucosa to adenoma, and finally to carcinoma (Arnold et al., 2017; Mutch, 2007). With the progression of COAD, gene mutations such as *TP53*, *Ras*, and *Braf*, and abnormal gene expressions including *TROP2*, *ABCG2*, and *HIF‐1*, have been reported to play crucial roles (Hall, Morris, & Sun, 2018; Recio‐Boiles & Cagir, 2019; Shawki, Ashburn, Signs, & Huang, 2018). However, the progression of COAD involving complex gene‐mechanisms has not yet been clearly explored.

Now many bioinformatics methods have been developed to identify potential prognostic biomarkers and therapeutic targets (Wan, Tang, Han, & Wang, 2018). For example, weighted gene co‐expression network analysis (WGCNA) can be used for finding clusters (or modules) of highly correlated genes (Langfelder & Horvath, 2008), for summarizing such clusters using the module eigengene or an intramodular hub gene, for relating modules to one another and to external sample traits, and for calculating module membership (MM) measures (Wan et al., 2018). WGCNA has been used to identify gene modules related to clinical outcomes in many cancers including colon cancer for evaluating recurrence (Ding, Li, Wang, Chi, & Liu, 2019). However, there is no study to systematically identify the regulatory network of transcription factor (TF)‐microRNA (miRNA)‐target gene related to the progression of COAD using WGCNA. miRNAs are small noncoding regulatory RNAs, which can bind to the target messenger RNAs (mRNAs) and repress the target mRNA expression. After regulated by TFs, miRNAs greatly affect the progression of COAD.

In this study, a co‐expression network of differentially expressed genes (DEGs) in COAD was constructed and the most significant modules in the network were used to reveal the tumorigenic genes. Then hub genes were identified by taking the intersection of DEGs between datasets GSE20916 and GSE39582 and validated using The Cancer Genome Atlas (TCGA) database. Finally, a regulatory network of TF‐miRNA‐target gene was established for the progression of COAD.

## 2 MATERIALS AND METHODS

### 2.1 Data preprocessing

The gene expression profiling datasets GSE20916 (https://www.ncbi.nlm.nih.gov/geo/query/acc.cgi?acc=GSE20916) and GSE39582 (https://www.ncbi.nlm.nih.gov/geo/query/acc.cgi) were downloaded from the Gene Expression Omnibus (GEO) database (Barrett et al., 2012). The GSE20916 dataset contains 44 normal, 55 adenoma, 36 adenocarcinoma, and 10 carcinoma tissue samples. The GSE39582 dataset contains 19 normal and 566 adenocarcinoma tissue samples. All samples are hybridized by Affymetrix Human Genome U133 Plus 2.0 Array. The matrix of the dataset was normalized by the limma package. The inclusion criteria of the GSE20916 included samples with normal, adenoma, and adenocarcinoma tissue types. The exclusion criteria of GSE39582 included the samples without disease‐free survival or the disease‐free survival time is 0.

### 2.2 Construction of WGCNA and identifying preserved modules

The GSE20916 matrix that contained gene expression (including normal, adenoma, and COAD samples) was ranked by the standard deviation (SD), and top 5,000 genes (Table S1) were selected for the next WGCNA analysis. Then the ideal soft‐thresholding power was chosen and genes cluster into modules was based on the Topological Overlap Matrix. The correlation between module eigengene and clinical characteristics was investigated by Pearson's correlation tests. *p* < .05 and |*r*| > .7 were considered as the selection criteria of candidate modules. Module preservation was calculated by the module preservation function to detect the stability and repeatability of modules in GSE39582 (Table S2). The module with maximum preservation statistics Zsummary and minimum preservation statistics medianRank was selected as the key module.

### 2.3 Function enrichment analysis

To investigate the potential biological function and pathways in the key module, the Online Database for Annotation, Visualization and Integrated Discovery (DAVID; https://david-d.ncifcrf.gov/) was used to analyze and visualize Gene Ontology (GO) terms and the Kyoto Encyclopedia of Genes and Genomes (KEGG) pathways (Table S3).

### 2.4 Screening hub genes

MM shows the correlation between genes and modules, and the top 15 genes with the highest correlation with the key module were selected as Group 1 of candidate genes. DEGs between normal colon tissue and adenocarcinoma in the GSE39582 dataset were determined by limma package based on the standard of adjusted *p* < .01 and | log FC | > 2. Then the disease free survival (DFS) of DEGs was calculated by survival package and genes with *p* < .05 were defined as Group 2 of candidate genes (Table S4). Hub genes were identified by the intersection of the two candidate gene groups.

### 2.5 Validating for hub genes

Gene Expression Profiling Interactive Analysis (GEPIA; http://gepia.cancer-pku.cn/) was used to analyze the RNA sequencing data from TCGA. TCGA data of COAD were used to validate the expression of identified hub genes. Human Protein Atlas (http://www.proteinatlas.org) was used to validate the candidate hub genes by immunohistochemistry.

### 2.6 Construction of TF regulatory network

StarBase (http://starbase.sysu.edu.cn/index.php) was used to predict miRNAs that bind to hub genes based on the standard that CLIP Data > = 3 and expression was present in at least one tumor sample. Then miRNAs with most intersections in 7 databases were selected (Fig. S3A‐G). The plugin iRegulon of Cytoscape is used to predict TF regulation networks.

## 3 RESULTS

### 3.1 Data preprocessing

A total of 145 samples in GSE20916 and 585 samples in GSE39582 were downloaded from GEO dataset and were normalized by the limma package. Then 135 samples from GSE20916 and 536 samples from GSE39582 were selected for the next step. Fig S1 shows the flow chart of the study design. Our selected clinical characteristics included histological results (normal colon epithelial tissue, adenoma, and adenocarcinoma), gender (male and female), and age.

### 3.2 Construction of WGCNA and identifying preserved modules

**Figure 1:**
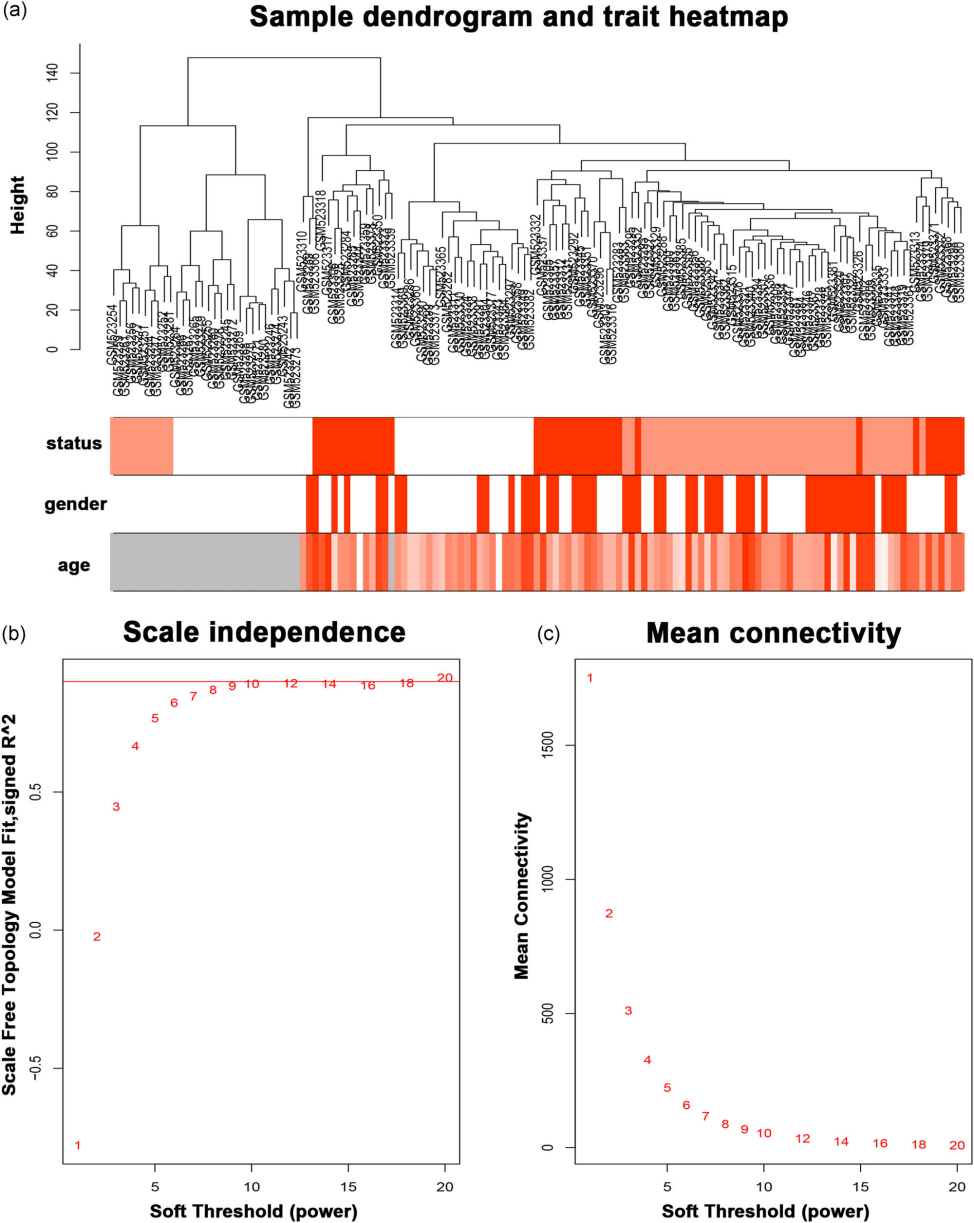
(a) The cluster was based on the expression data of GSE20916, which contained 44 normal tissue samples, 55 adenoma tissue samples, and 36 adenocarcinoma tissue samples. The top 5,000 genes with the highest SD values were used for the analysis by WGCNA. The color intensity was proportional to disease status (normal, adenoma, and adenocarcinoma), sex (male and female) and age (years old). (b) Analysis of the scale‐free fit index for various soft‐thresholding power (β). Analysis of the mean connectivity for various soft‐thresholding power. In all, 10 was the most fit power value. SD, standard deviation

**Figure 2:**
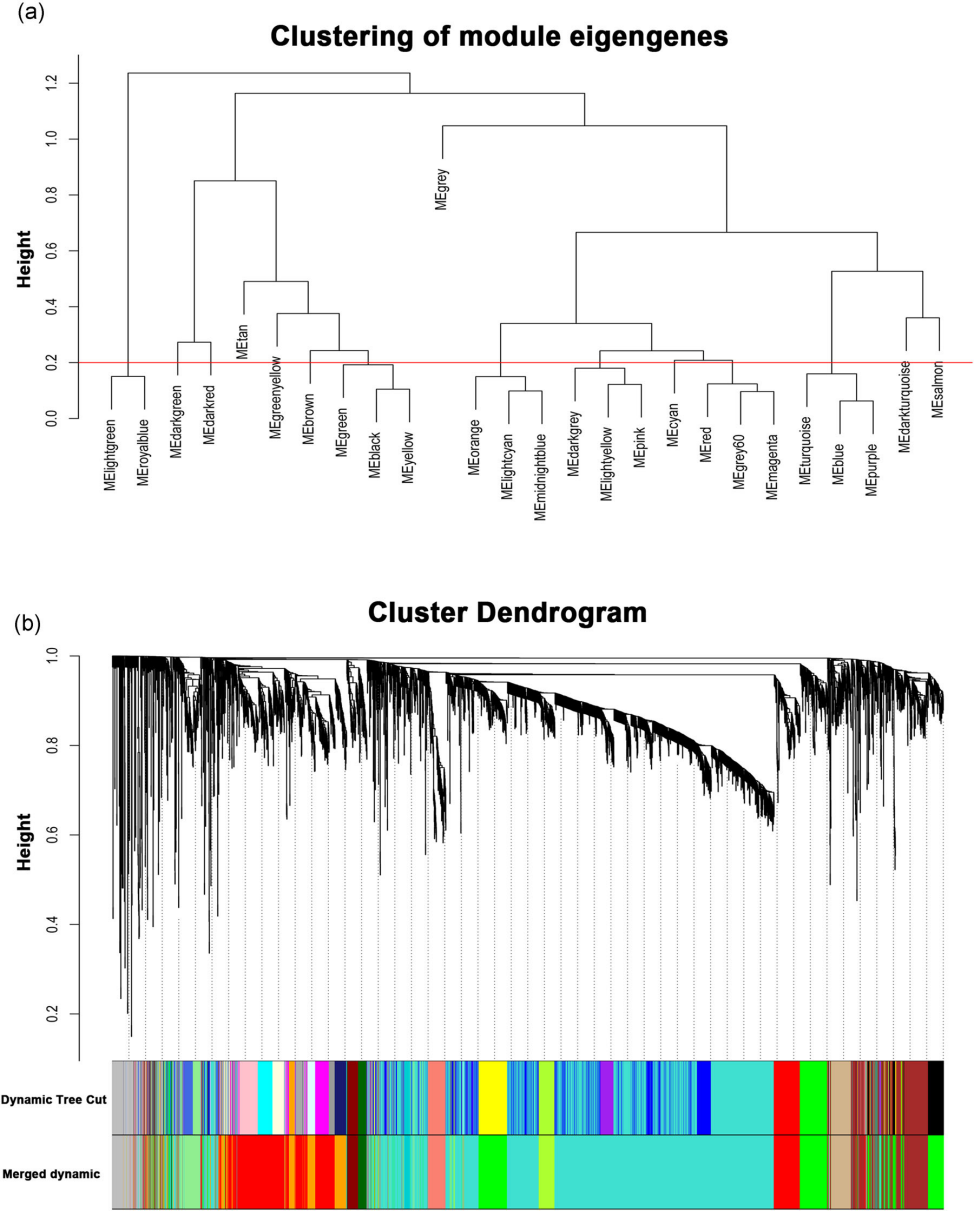
(a) The cluster dendrogram of module eigengenes. (b) The cluster dendrogram of genes in GSE20916. Each branch in the figure represented one gene, and every color below represented one co‐expression module

**Figure 3:**
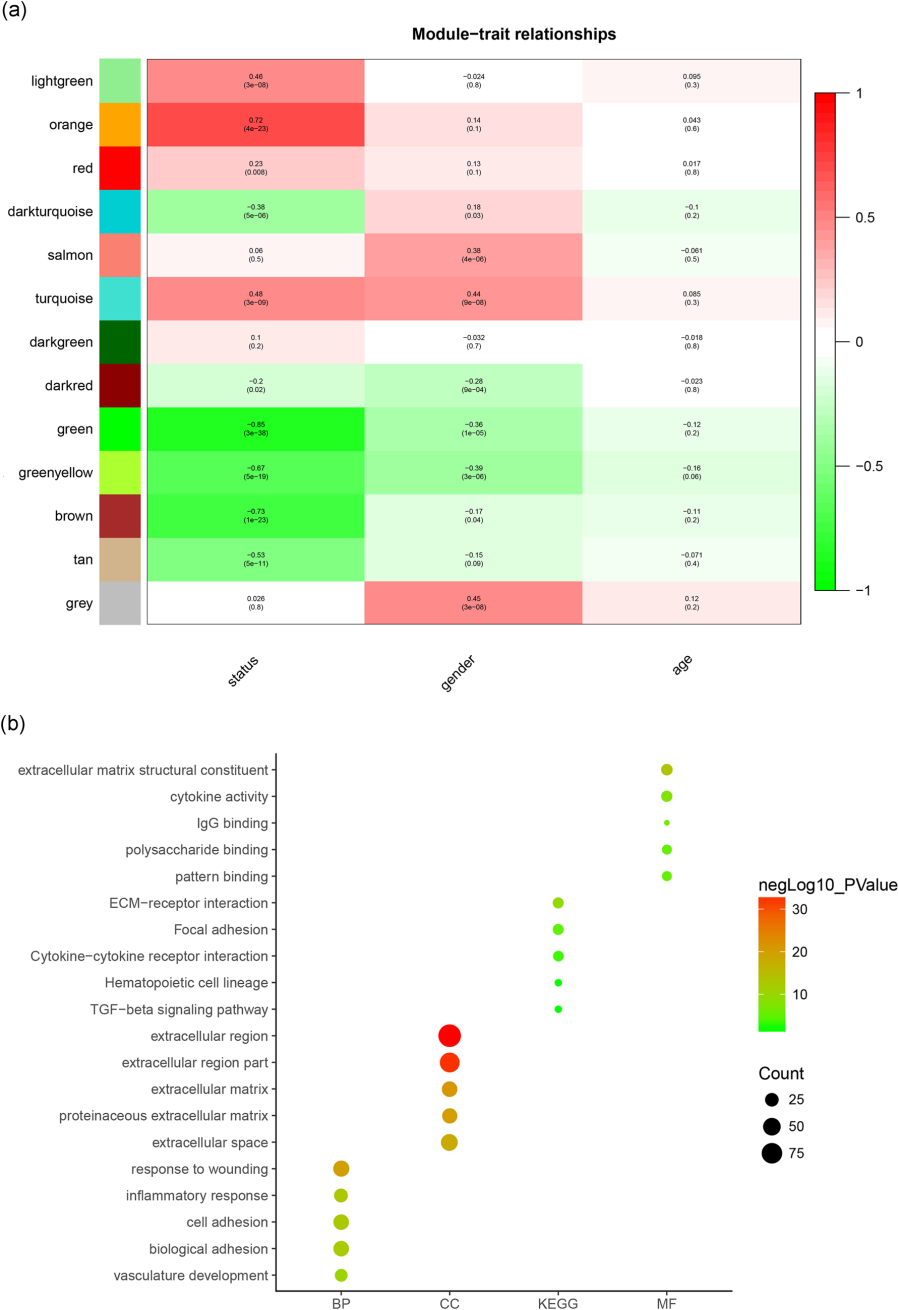
(a) Heat‐map of the correlation between module eigengenes and the disease status of colon adenocarcinoma. The orange module was most positively correlated with status. (b) Bubble chart of GO and KEGG results of orange module. GO, Gene Ontology; KEGG, Kyoto Encyclopedia of Genes and Genomes

**Figure 4:**
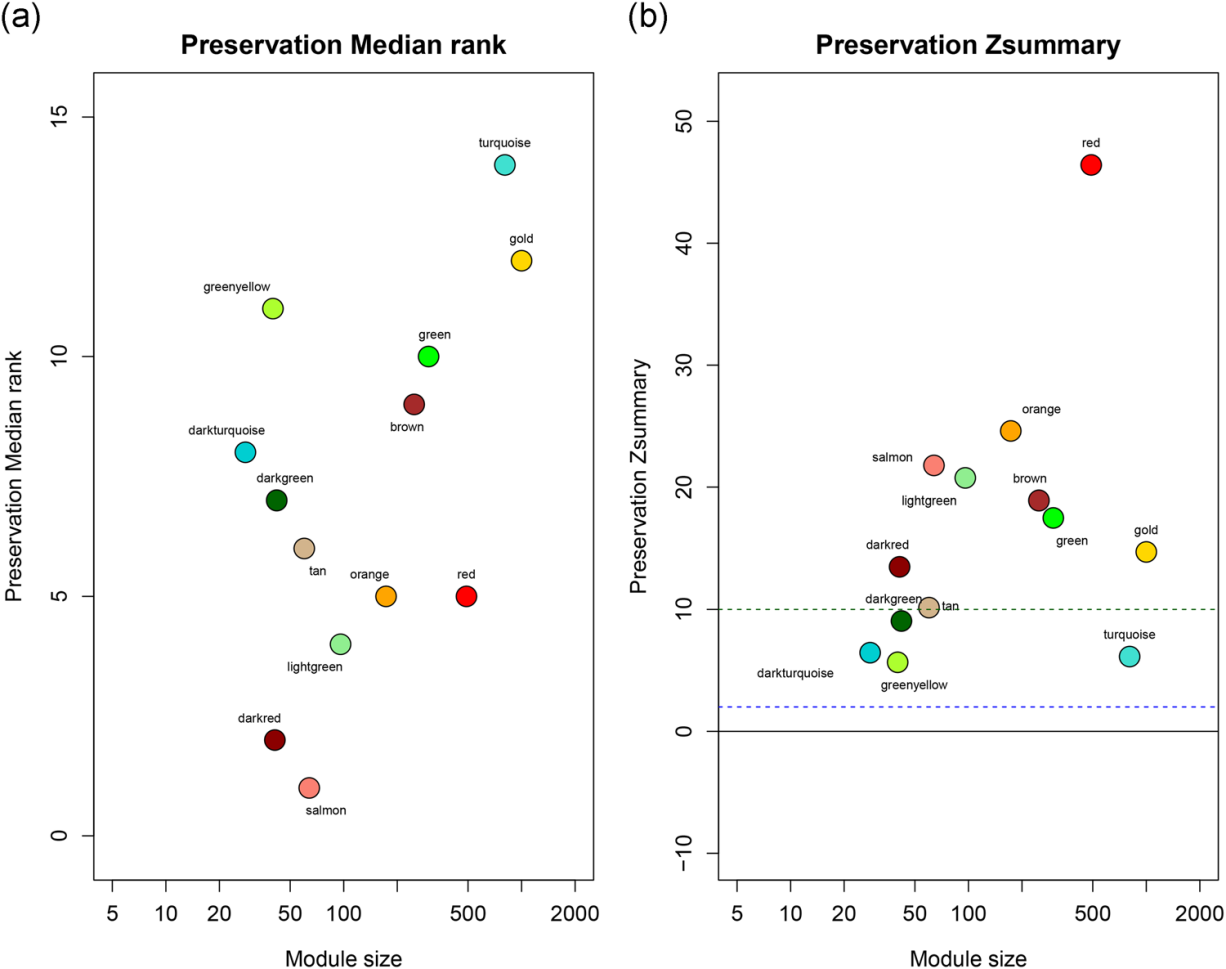
Module preservation was evaluated by medianRank and Zsummary statistics which correlated to connectivity and density of networks. The module with lower medianRank tends to exhibit stronger observed preservation than the module with a higher medianRank if both of them are preserved. Compared with the GSE20916 dataset, orange module with highest Zsummary (a)and lowest medianRank statistics (b) than brown and green modules

The sample dendrogram was plotted using the WGCNA package (Figure 1a). The appropriate soft‐thresholding power was chosen with the ideal soft‐thresholding power of 10 (Figure 1b). Twenty‐six modules were identified by the Hierarchical Clustering and the Dynamic branch Cutting based on the minimum module size 30. Then 26 modules merge in 13 modules based on the similarity between module eigengenes above 0.8 (Figure 2). Further, the correlation between modules eigengenes and histological results (normal colon epithelial tissue, adenoma, and adenocarcinoma) was analyzed. There were four modules positively associated with histological results, and six modules negatively associated (Figure 3a; *p* < .01). Among these modules, brown, green, and orange modules had the highest correlations with histological results (Figure 3a; all |*r*| > .7), indicating that they play important roles in the tumorigenesis of COAD. Expression of 5,000 genes with the largest difference of SD in dataset GSE39582 was used to calculate the preservation of modules. Figure 4a,b shows the medianRank statistics and Zsummary statistics of each module. Among the brown, green, and orange modules with preservation, the orange module with the maximum statistics Zsummary and minimum statistics medianRank was defined as the key module.

### 3.3 Enrichment analysis of the orange module

The online database of DAVID was used to explore the enrichment analysis of GO and KEGG in key orange module. Figure 3b shows the enrichment results of GO and KEGG in the orange module. The KEGG of orange module was mainly in the extracellular matrix receptor (ECM)–receptor interaction, focal adhesion, cytokine–receptor interaction, hematopoietic cell lineage, and transforming growth factor (TGF)‐β signaling pathway, while the biological process based on GO of orange module mainly included response to wounding, inflammatory response, cell adhesion, biological adhesion, and vasculature development. Figure 3b show the contents of cellular component and molecular function based on the GO of orange module.

### 3.4 Identification and validation of hub genes

**Figure 5:**
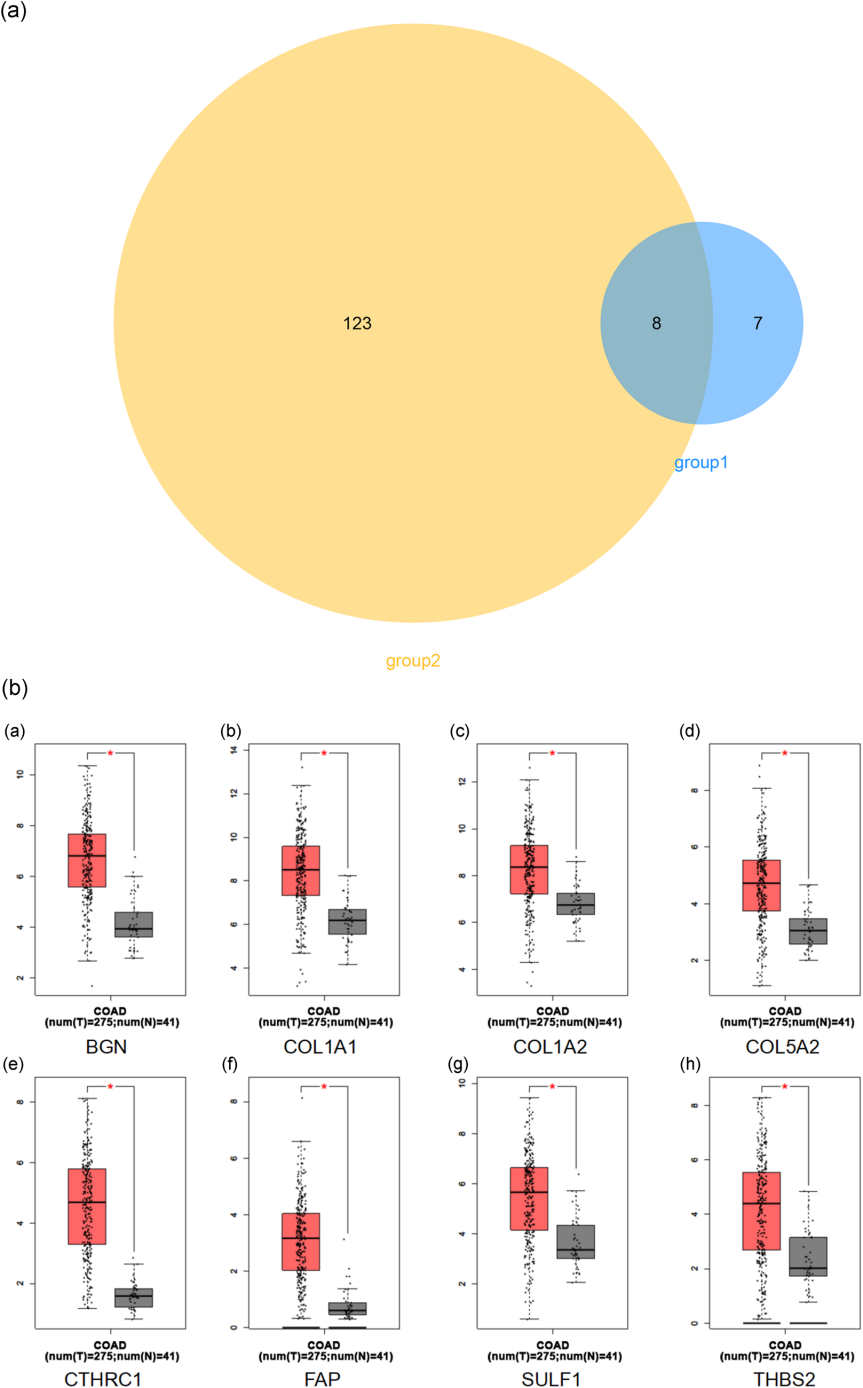
(a) Venn plot of candidate hub genes commonly owned in GS20916 and GSE39582. (b) The transcriptional differences of hub gene levels between colon carcinoma tissues and the para‐cancer tissues in TCGA. TCGA, The Cancer Genome Atlas (*p < .001)

**Figure 6:**
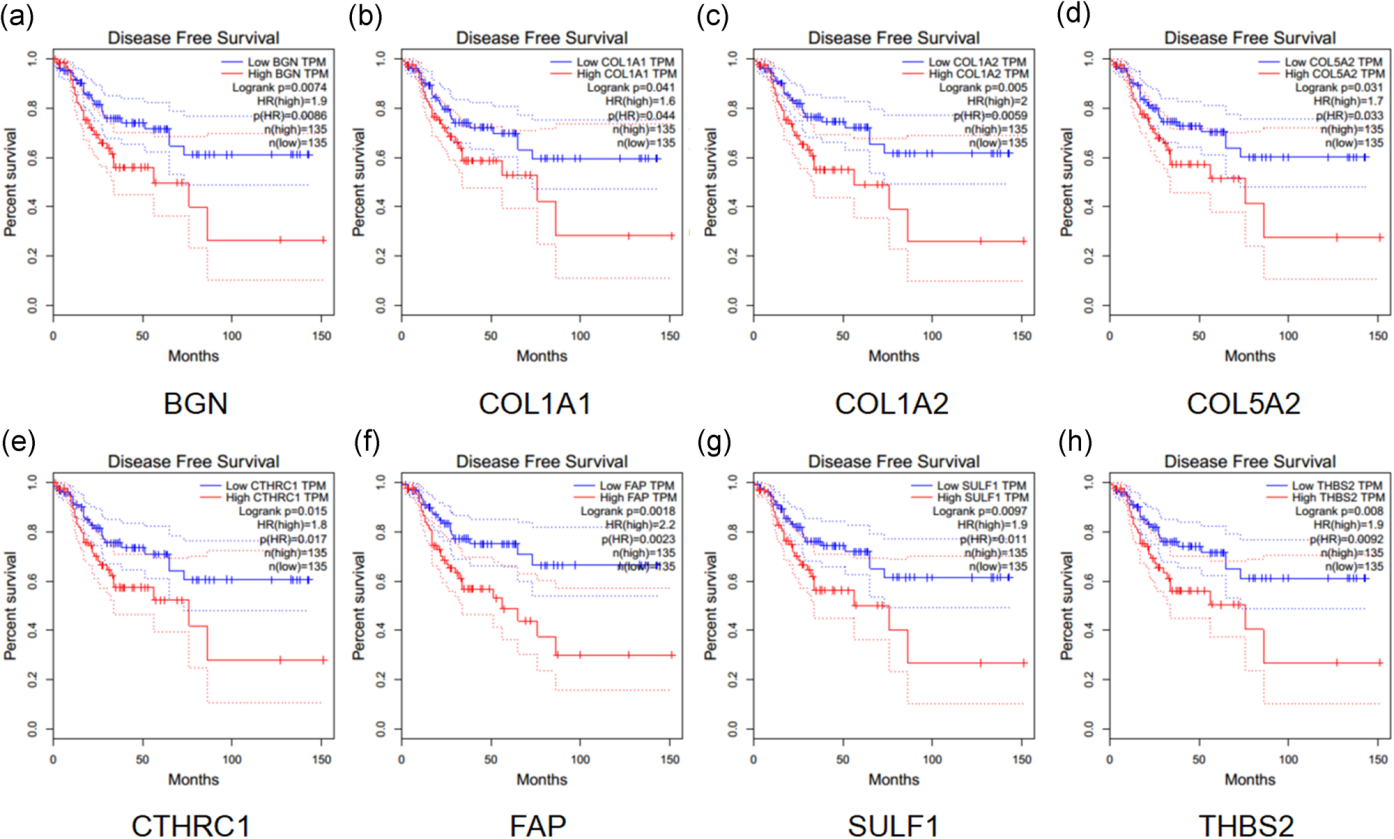
The disease‐free survival analysis of hub gene levels in colon carcinoma patients in TCGA. TCGA, The Cancer Genome Atlas

**Figure 7:**
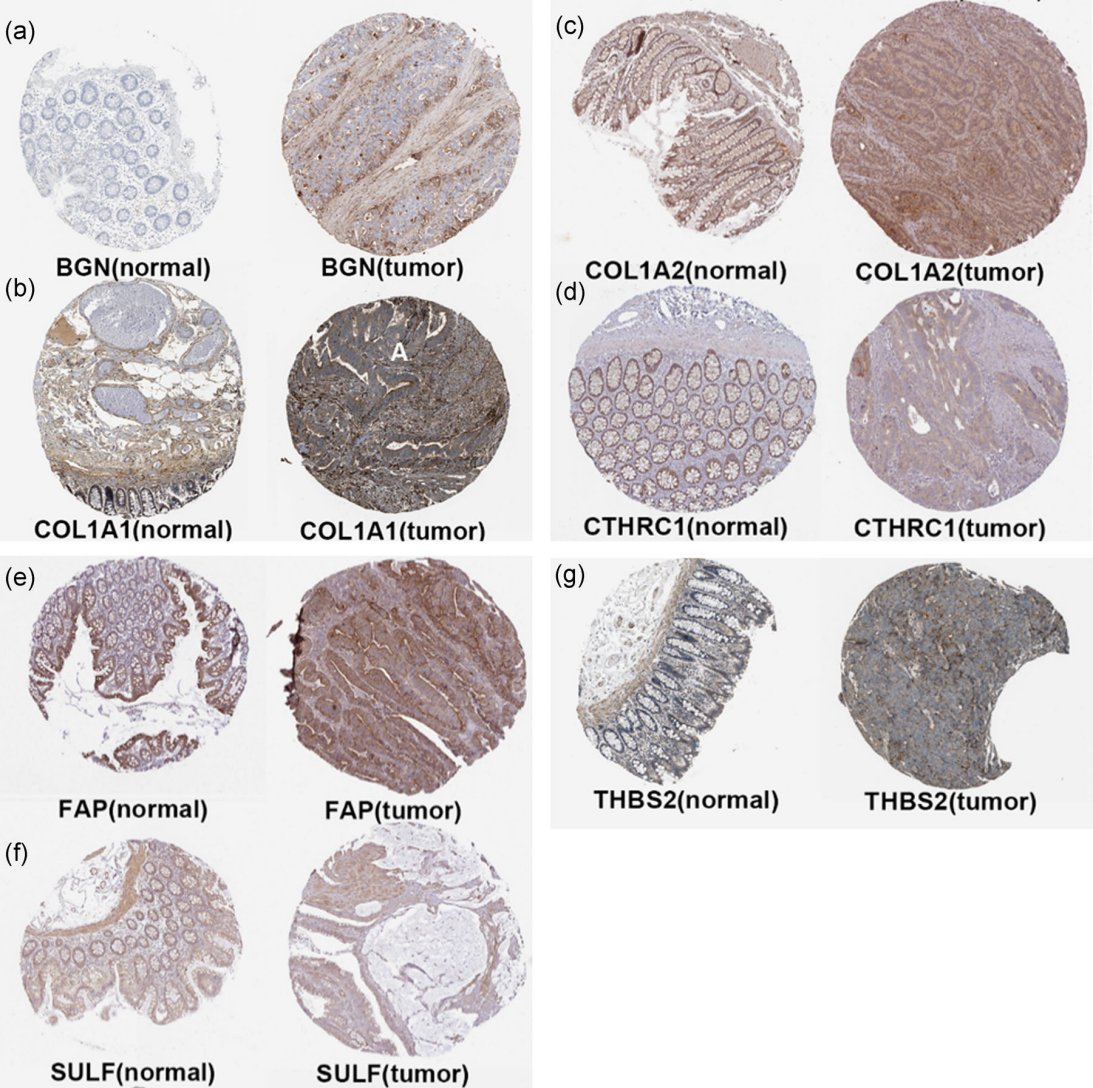
The translational differences of hub gene levels between colon carcinoma tissues and the paracancer tissues in the Human Protein Atlas database

A total of eight genes (*BGN*, *SULF1*, *COL1A1*, *FAP*, *THBS2*, *CTHRC1*, *COL5A2*, and *COL1A2*) shared by both Groups 1 and 2 of candidate genes were identified as hub genes (Figure 5a). We used TCGA data of COAD to validate the hub gene expression with the online tool of GEPIA. All of the hub genes are expressed differently in normal and cancer tissues of COAD by the criterion of |logFC| > 1 and *p* < .01 (Figure 5b). All of the hub genes significantly related to disease‐free survival of COAD (Figure 6) and GSE39582 (Figure S2). Compared to normal tissues, the protein expressions of these hub genes in tumor tissues were significantly higher based on the Human Protein Atlas database (Figure 7).

### 3.5 Construction of TF regulatory network

**Figure 8:**
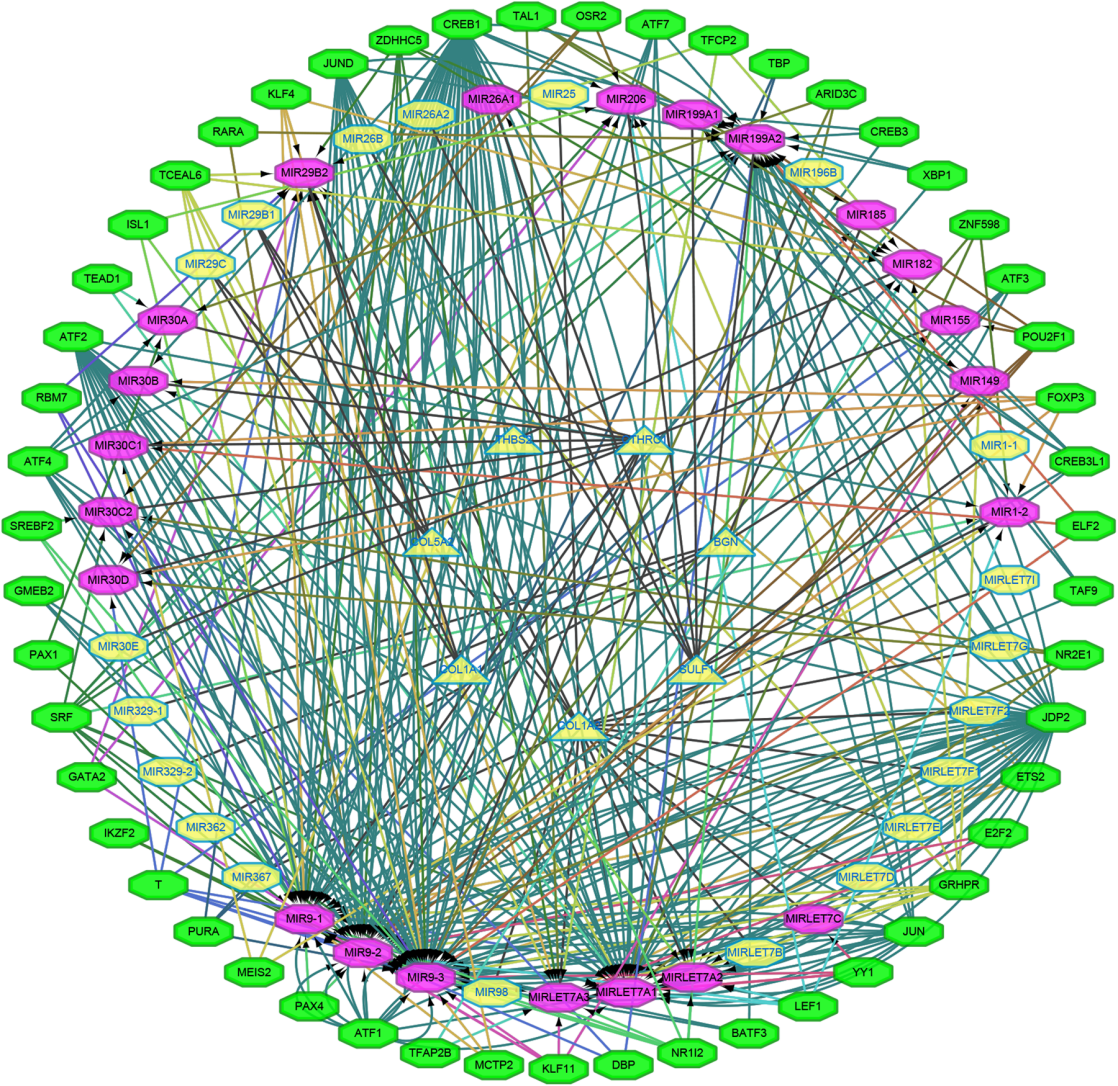
The transcriptional regulatory network of hub genes, miRNAs, and TFs. miRNAs, microRNAs; TFs, transcription factors

A total of 42 miRNAs could bind to hub genes predicted by starBase. One hub gene *FAP* does not have any binding miRNA. The related TF and the TFs regulatory network are shown in Figure 8. The regulatory network of TF‐miRNA‐target gene was established, involving 144 TFs, 26 miRNAs, and 7 hub genes, such as model KLF11‐miR149‐*BGN*, TCEAL6‐miR29B2‐*COL1A1*, TCEAL6‐miR29B2‐*COL1A2*, TCEAL6‐miR29B2‐*COL5A2*, TEAD1‐miR30A‐*CTHRC1*, CREB3‐miR199A1‐*SULF1*, and GRHPR‐miR182‐*THBS2*.

## 4 DISCUSSION

High‐throughput biological technologies, such as the systems biology algorithm of WGCNA, allow researchers to evaluate the relationships between genes and clinical features through a scale‐free network (Langfelder & Horvath, 2008; Wan et al., 2018). WGCNA has been widely used in disease, physiology, drug, genome annotation, and cancer progression (Chen et al., 2018). WGCNA has been used to identify gene modules and hub genes associated with TNM stages in gastric cancer, hepatocellular carcinoma (Zhu, Sun, Zhou, He, & Qian, 2018), and metastatic colorectal cancer (Wang et al., 2014). In this study, datasets GSE20916 and GSE39582 were utilized to construct co‐expression networks by WGCNA to identify the key orange module related to the progression of COAD.

Herein, we found eight hub genes for the progression of COAD, including *BGN*, *SULF1*, *COL1A1*, *FAP*, *THBS2*, *CTHRC1*, *COL5A2*, and *COL1A2*. These genes may contribute to the exploration of the progression of COAD and could be regarded as biomarkers and therapeutic targets of COAD. Most of these genes have been proved to relate to the tumorigenesis and metastasis of cancer. *BGN* is an extracellular matrix protein which plays vital roles in the regulation of cell morphology, growth, migration, and differentiation. The abnormal expressions of *BGN* in ovarian cancer and osteosarcoma were closely associated with chemoresistance. In HCT116 colon cancer cell line, *BGN* knockdown inhibited the proliferation and invasion (Xing, Gu, & Ma, 2015). Furthermore, *BGN* has been reported to promote the chemotherapy resistance via activating nuclear factor kappa‐light‐chain‐enhancer of activated B cells signal transduction in colon cancer (Liu, Xu, Xu, Cui, & Xing, 2018). The *SULF1* gene encoded the heparin‐degrading endo‐sulfatase to inhibit the activation of cell growth factor. The expression of *SULF1* is stable in normal tissues but downregulated in cancer cells. Moreover, cancer cell proliferation and migration could be inhibited by re‐expression of *SULF1* (Lai, Sandhu, Shire, & Roberts, 2008). The activation of the TGFβ/SMAD transcriptional pathway underlays a novel tumor‐promoting role of *SULF1* in hepatocellular carcinoma (Dhanasekaran et al., 2015). *COL1A1* encodes the pro‐α 1 chains of type I collagen. *COL1A1* was reported to be overexpressed in many cancers (Shi et al., 2019). *COL1A1* could promote colorectal cancer metastasis by regulating the WNT/PCP pathway (Zhang, Wang, Zhang, Zhong, & Yang, 2018). *THBS2* is a thrombospondin family protein and negatively regulates angiogenesis in cancer. Increasing the *THBS2* expression in cancer inhibited tumor growth (Sun et al., 2014). *THBS2* expression in colorectal cancer was associated with reduced angiogenesis and distant metastasis (Wang et al., 2016). Most recently, miR‐135b was reported to promote invasion and metastasis through *THBS2* suppression (Nezu et al., 2016). Higher *THBS2* expression in colorectal cancer tissue was found compared with normal tissue (Wang et al., 2016). Furthermore, *RBP4* and *THBS2* have been reported to serve as biomarkers for the diagnosis of colorectal cancer (Fei et al., 2017).

In addition, *CTHRC1* is an extracellular matrix glycoprotein and regulates the Wnt‐PCP pathway in developmental morphogenesis (Ma et al., 2014). Recently, *CTHRC1* was found to be highly expressed in many human cancers, promoting invasion and metastasis (Tan et al., 2013). *CTHRC1* has been reported to promote proliferation and invasiveness of human colorectal cancer cells by activating Wnt/PCP signaling (Yang et al., 2015). *COL5A2*, also known as collagen type V alpha 2 chain, involves in the progression of colorectal cancer, breast cancer, and osteosarcoma. *COL5A2* was reported to be correlated with poor clinical outcomes of bladder cancer patients, suggesting that it could serve as a cancer biomarker (Zeng, Liu, Liu, & Wang, 2018). *COL5A2* expression was also found in colorectal cancer but not in normal colon, showing that *COL5A2* was implicated in the carcinogenesis of colorectal cancer (Fischer, Stenling, Rubio, & Lindblom, 2001). Type I collagen consists of one α2 chain (*COL1A2*) and two α1 chains (*COL1A1*). *COL1A2* was downregulated in many cancers, including melanoma, bladder cancer, and head and neck cancer. However, in pancreatic and ovarian cancer, *COL1A2* was upregulated. The different expressions of *COL1A2* cause distinct collagen‐mediated effects in different human malignancies (Ao, Guan, Wang, & Wang, 2018; Wu et al., 2019). Upregulation of *COL1A2* was reported in blood and tumor tissues of patients with colorectal cancer (Rodia et al., 2018). Inhibition of *COL1A2* expression in colorectal cancer cell could suppress cell proliferation and invasion (Yu et al., 2018).

In our study, Cytoscape and StarBase were used to construct a regulation network of TF‐miRNA‐mRNA. Although miRNAs like miR‐155‐5p, miR‐199a‐5p and miR‐206 were clearly associated with the development and progression of colon cancer, miRNAs associated with colon cancer are still not thoroughly explored (Parasramka et al., 2012; Qu et al., 2015; Sakurai et al., 2011). We established the first regulatory network of TF‐miRNA‐target gene for the progression of COAD, involving 144 TFs, 26 miRNAs, and 7 hub genes, such as model KLF11‐miR149‐*BGN*, TCEAL6‐miR29B2‐*COL1A1*, and TCEAL6‐miR29B2‐*COL1A2*.

In summary, we found a total of eight hub genes (*BGN*, *SULF1*, *COL1A1*, *FAP*, *THBS2*, *CTHRC1*, *COL5A2*, and *COL1A2*) closely related to the survival of patients with COAD. These genes and factors may contribute to the exploration of the progression of COAD and could be regarded as biomarkers and therapeutic targets of COAD. A regulatory network of TF‐miRNA‐target gene was established, involving 144 TFs, 26 miRNAs, and 7 hub genes, which could help to understand disease progression and optimize treatment strategy.

## CONFLICT OF INTERESTS

The authors declare that there are no conflict of interests.

## AUTHOR CONTRIBUTIONS

Z. H. and X. L. contributed to the conception or design of the work. S. W., Y. H., and J. C. contributed to acquisition, analysis, and interpretation of data for the work. Q. S., H. W., and X. L. contributed to manuscript preparation, revision, and review.

## Supporting information

Supporting information

Supporting information

Supporting information

Supporting information

Supporting information

## Data Availability

The data that support the findings of this study are available in Gene Expression Omnibus (GEO) datasets at https://www.ncbi.nlm.nih.gov/gds/.
