# Supplementary material for: Identification of hub genes and construction of transcriptional regulatory network for the progression of colon adenocarcinoma hub genes and TF regulatory network of colon adenocarcinoma
Source: J Cell Physiol. 2019 Oct 14;235(3):2037–48. doi: 10.1002/jcp.29067 (PMC6916361; doi:10.1002/jcp.29067)
Supplement: Supplementary file 1 — Supporting information [file JCP-235-2037-s001.pptx]

## Slide 1
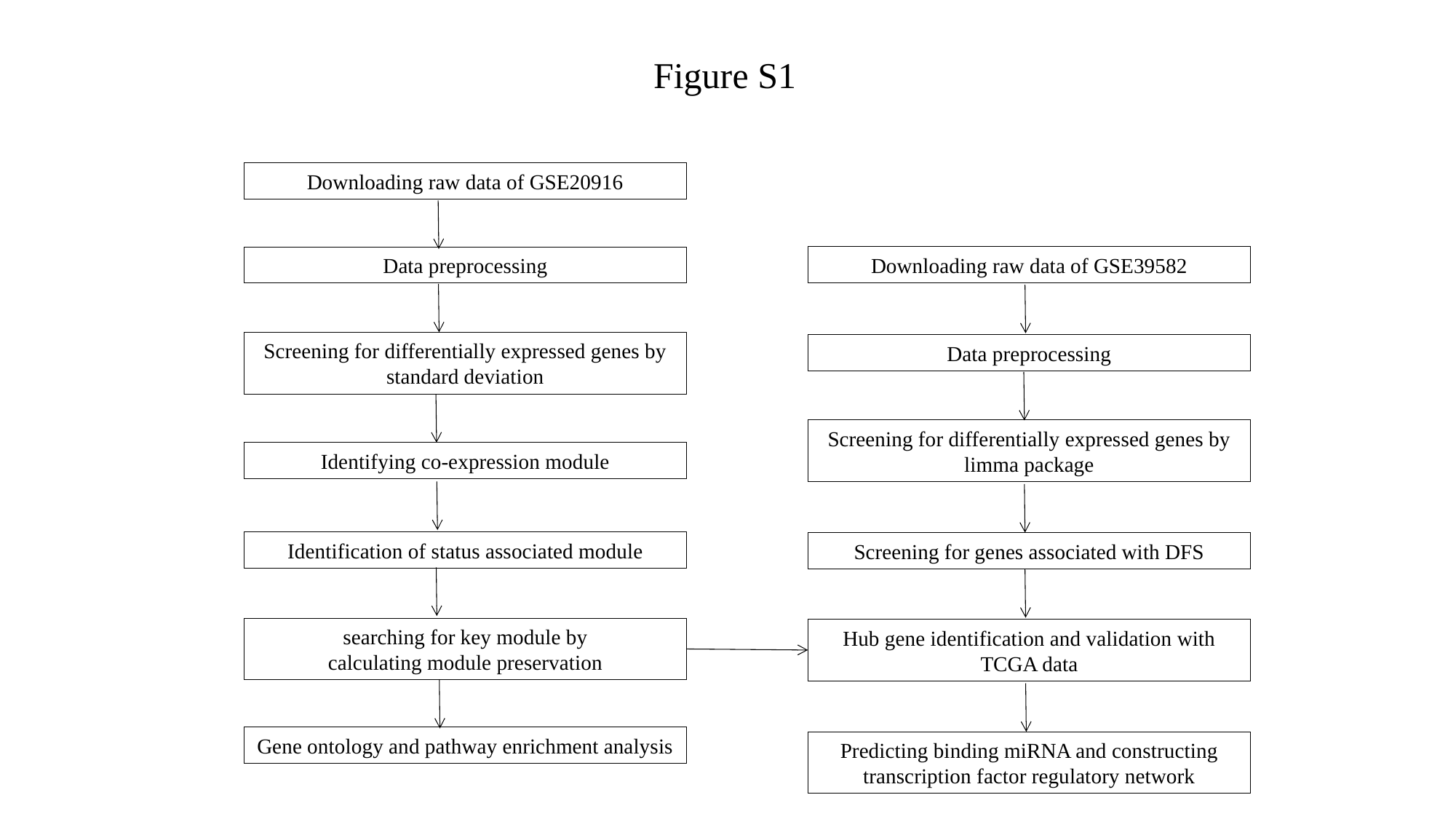

Figure S1
Downloading raw data of GSE20916
Downloading raw data of GSE39582
Data preprocessing
Screening for differentially expressed genes by standard deviation
Data preprocessing
Screening for differentially expressed genes by limma package
Identifying co-expression module
Identification of status associated module
Screening for genes associated with DFS
searching for key module by
calculating module preservation
Hub gene identification and validation with TCGA data
Gene ontology and pathway enrichment analysis
Predicting binding miRNA and constructing transcription factor regulatory network

## Slide 2
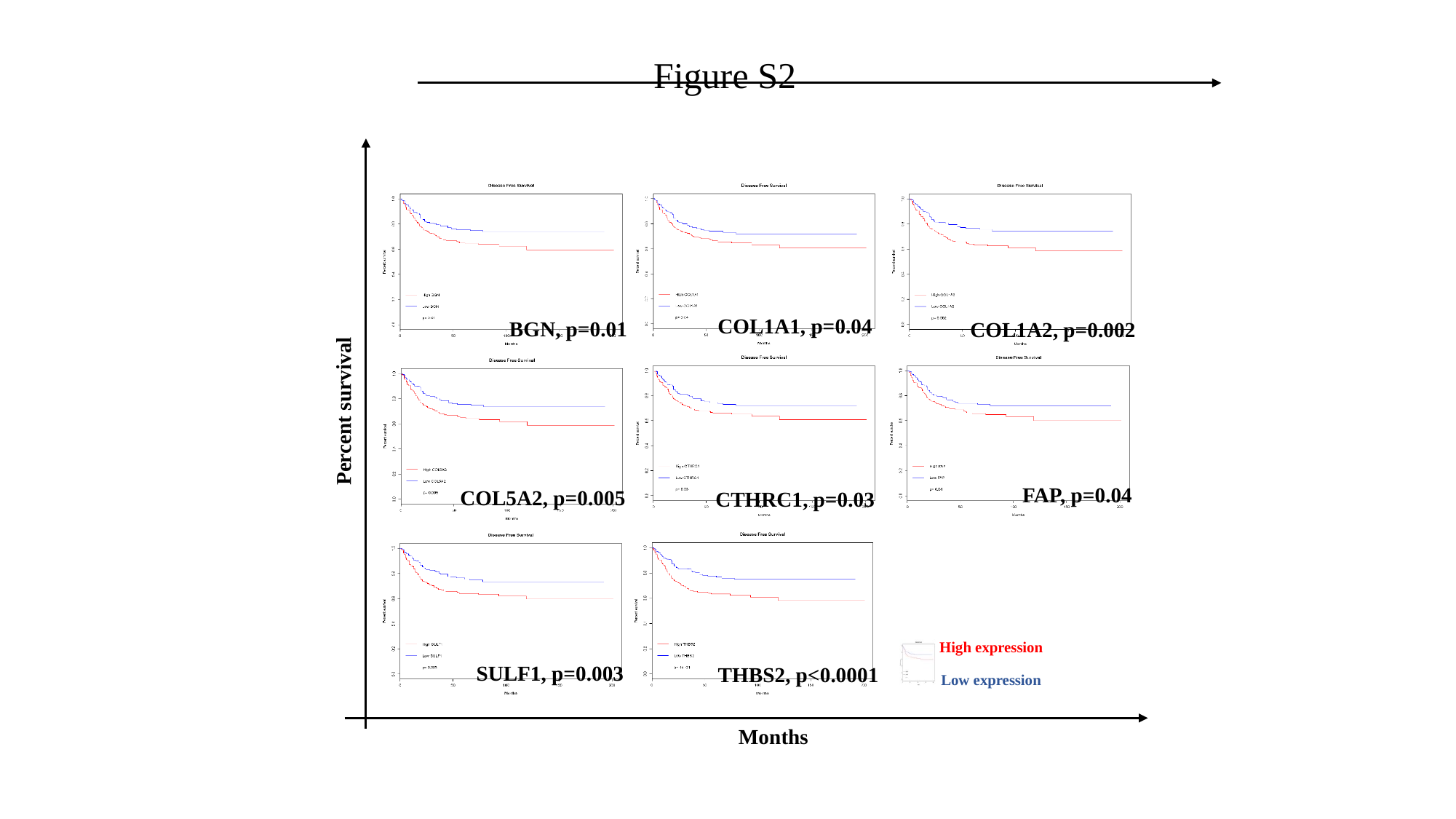

Figure S2
COL1A1, p=0.04
BGN, p=0.01
COL1A2, p=0.002
Percent survival
FAP, p=0.04
COL5A2, p=0.005
CTHRC1, p=0.03
High expression
SULF1, p=0.003
THBS2, p<0.0001
Low expression
Months

## Slide 3
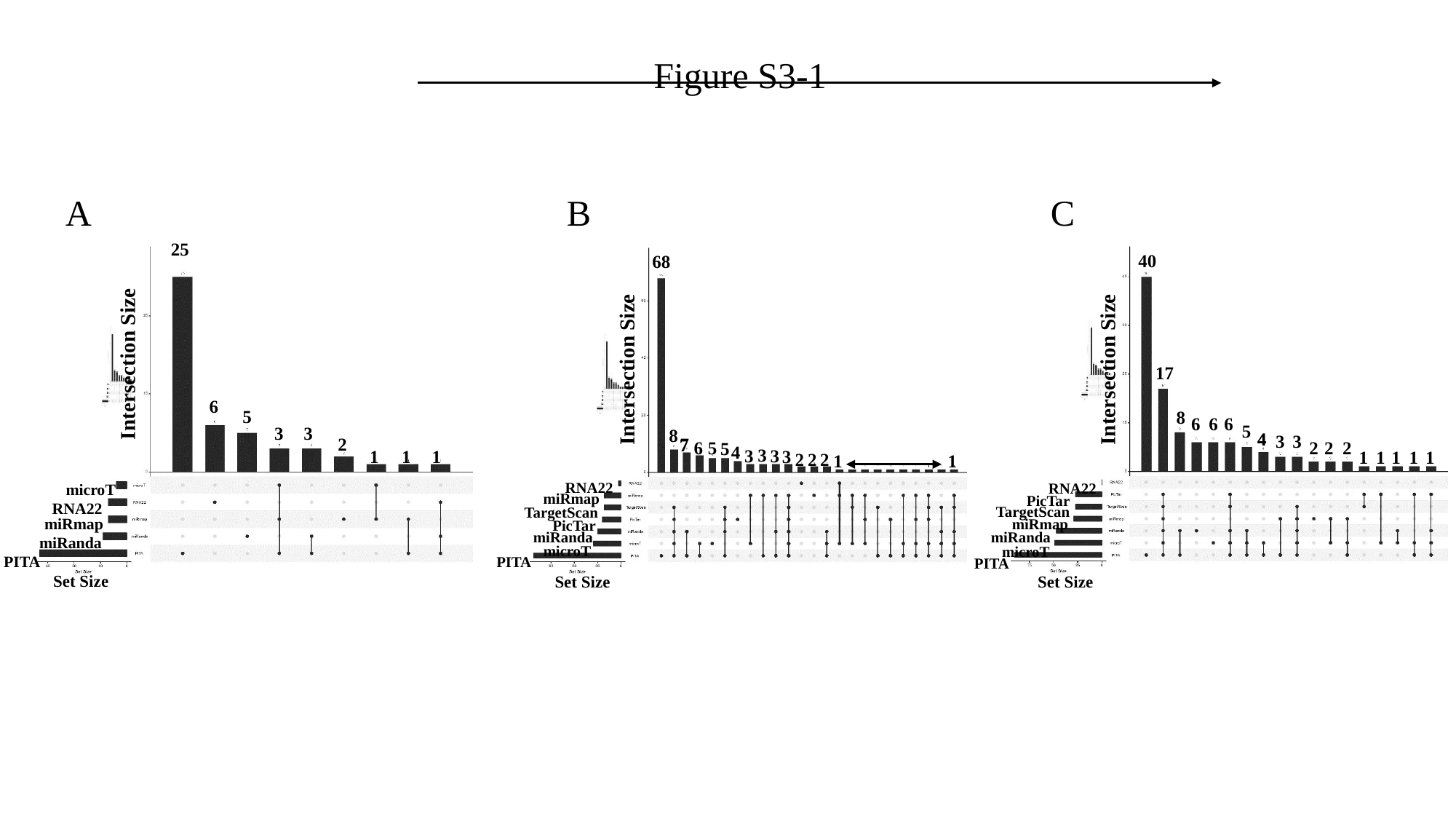

Figure S3-1
A
C
B
25
40
68
Intersection Size
Intersection Size
Intersection Size
17
6
5
8
6
6
6
5
3
3
8
4
4
3
3
2
7
7
2
2
6
5
2
5
4
3
3
3
1
1
3
1
1
1
1
1
1
2
2
2
1
1
RNA22
RNA22
microT
miRmap
PicTar
RNA22
TargetScan
TargetScan
miRmap
miRmap
PicTar
miRanda
miRanda
miRanda
microT
microT
PITA
PITA
PITA
Set Size
Set Size
Set Size

## Slide 4
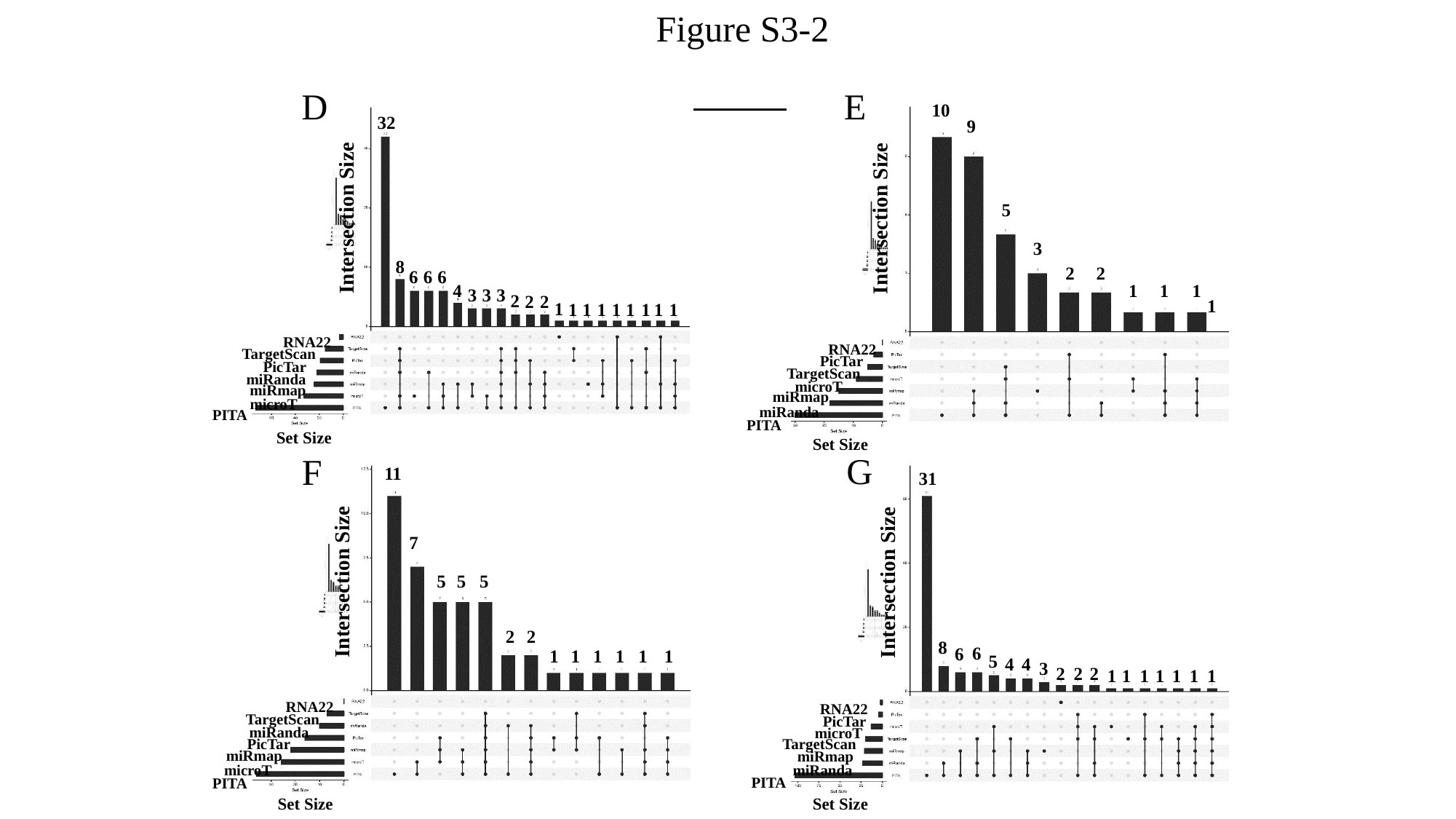

Figure S3-2
E
D
10
32
9
5
Intersection Size
Intersection Size
3
8
2
2
6
6
6
4
1
1
1
3
3
3
2
2
2
1
1
1
1
1
1
1
1
1
1
RNA22
RNA22
TargetScan
PicTar
PicTar
TargetScan
miRanda
microT
miRmap
miRmap
microT
miRanda
PITA
PITA
Set Size
Set Size
G
F
11
31
7
Intersection Size
5
Intersection Size
5
5
2
2
8
6
6
1
1
1
1
1
1
5
4
4
3
2
2
2
1
1
1
1
1
1
1
RNA22
RNA22
TargetScan
PicTar
miRanda
microT
PicTar
TargetScan
miRmap
miRmap
miRanda
microT
PITA
PITA
Set Size
Set Size
